# Leber congenital amaurosis caused by *Lebercilin* (*LCA5*) mutation: Retained photoreceptors adjacent to retinal disorganization

**Published:** 2009-06-02

**Authors:** Samuel G. Jacobson, Tomas S. Aleman, Artur V. Cideciyan, Alexander Sumaroka, Sharon B. Schwartz, Elizabeth A.M. Windsor, Malgorzata Swider, Waldo Herrera, Edwin M. Stone

**Affiliations:** 1 Department of Ophthalmology, Scheie Eye Institute, University of Pennsylvania, Philadelphia, PA; 2 Howard Hughes Medical Institute and Department of Ophthalmology, University of Iowa Hospitals and Clinics, Iowa City, IA

## Abstract

**Purpose:**

To determine the retinal disease expression in the rare form of Leber congenital amaurosis (LCA) caused by *Lebercilin (LCA5)* mutation.

**Methods:**

Two young unrelated LCA patients, ages six years (P1) and 25 years (P2) at last visit, both with the same homozygous mutation in the *LCA5* gene, were evaluated clinically and with noninvasive studies. En face imaging was performed with near-infrared (NIR) reflectance and autofluorescence (AF); cross-sectional retinal images were obtained with optical coherence tomography (OCT). Dark-adapted thresholds were measured in the older patient; and the transient pupillary light reflex was recorded and quantified in both patients.

**Results:**

Both *LCA5* patients had light perception vision only, hyperopia, and nystagmus. P1 showed a prominent central island of retinal pigment epithelium (RPE) surrounded by alternating elliptical-appearing areas of decreased and increased pigmentation. Retinal laminar architecture at and near the fovea was abnormal in both patients. Foveal outer nuclear layer (ONL) was present in P1 and P2 but to different degrees. With increasing eccentricity, there was retinal laminar disorganization. Regions of pericentral and midperipheral retina in P1, but not P2, could retain measurable ONL and less laminopathy. P2 had a small central island of perception with >5 log units of sensitivity loss. Pupillary responsiveness was present in both *LCA5* patients; the thresholds were abnormally elevated by ≥5.5 log units.

**Conclusions:**

*LCA5* patients had evidence of retained photoreceptors mainly in the central retina. Retinal remodeling was present in pericentral regions in both patients. The NIR reflectance and NIR-AF imaging in the younger patient suggested preserved RPE in retinal regions with retained photoreceptors. Detailed phenotype studies in other *LCA5* patients with longitudinal follow-up will help determine the feasibility of future intervention in this rare disease.

## Introduction

Leber congenital amaurosis (LCA) is a molecularly heterogeneous retinal disease with visual impairment from early life [1,2]. Gene identification, proof-of-concept and safety studies in animals, and detailed human studies of photoreceptor layer integrity have led to treatment trials in the molecular form of LCA caused by mutations in *RPE65*, the gene encoding retinal pigment epithelium-specific protein, 65 kDa [3-10]. For other molecular forms of LCA, progress toward human clinical treatment trials is not as far along. One step that can be taken now, however, is to determine the treatment potential of the human diseases using detailed study of molecularly clarified LCA patients. To date, we have conducted studies with this goal in LCA caused by mutations in *CEP290* (Centrosomal protein, 290 kDa), *CRB1* (Crumbs homolog-1), *CRX* (Cone-rod homeobox-containing gene), *GUCY2D* (Guanylate cyclase 2D, retinal), *RDH12* (Retinol dehydrogenase 12), *RPE65*, *RPGRIP1* (Retinitis pigmentosa GTPase regulator-interacting protein), or *TULP1* (Tubby-like protein 1) [6,11-19].

The *LCA5* locus was initially mapped to chromosome 6p14.1 [20,21] and more recently the gene was determined to encode a protein, lebercilin, involved with photoreceptor ciliary function [22,23]. *LCA5* accounts for about 1%–2% of LCA. An animal model for *LCA5* that will permit further investigations of disease mechanism is awaited [1]. The literature that identified *LCA5* genotypes has included clinical descriptions of the disease. There is consensus that *LCA5* mutations lead to an early and severe visual disturbance with nystagmus, abnormal visual acuity, nondetectable electroretinograms, and fundus features of retinal degeneration [21,22,24,25]. Further details of retinal phenotype will be needed to define the potential for any future therapeutic approach to this rare disorder. We used clinical evaluation and retinal imaging modalities to study two LCA patients with the same homozygous *LCA5* mutation. Dark-adapted psychophysics and pupillometry were used to assay visual function [26,27].

## Methods

### Participants

Two LCA patients with *LCA5* mutations underwent complete eye examinations and studies of retinal phenotype. For comparison with the *LCA5* results, we included 30 patients (ages 1–59) who had other forms of LCA or retinal degenerations not considered LCA. Also studied were 34 normal participants (ages 5–58). Informed consent was obtained from all participants. The study procedures followed the Declaration of Helsinki and were approved by the institutional review board of the University of Pennsylvania.

### En face imaging with scanning laser ophthalmoscope

Near-infrared (NIR) light was used to perform en face imaging with a confocal scanning laser ophthalmoscope (SLO; HRA2, Heidelberg Engineering GmbH, Heidelberg, Germany) without subjecting the diseased retina to undue light exposure [28]. Reflectivity distribution of retinal and subretinal features was imaged with 820 nm NIR light. Health of the RPE was estimated with NIR-autofluorescence (NIR-AF) using 790 nm excitation light and a long-pass blocking filter that allowed detection of fluorescence emissions of >810 nm [16,28-31]. NIR-AF signal is believed to be dominated by the melanolipofuscin in RPE and melanin in the RPE and choroid [16,28-36]. Disease-related changes in RPE melanin content result in spatial variation of the NIR-AF signal intensity, appearance, or both. Imaging was performed in the high-speed mode; 30°×30° of retina was sampled onto a 768×768 pixel image, and video segments of up to 10 s length were obtained at the rate of 8.8 Hz. Detector sensitivity was set to 95% for NIR-AF. Automatic real-time (ART) averaging feature of the manufacturer’s software was used whenever possible. When ART failed, images were exported from the manufacturer’s software and analyzed as previously described [16,28-31].

### Optical coherence tomography

Retinal cross-sections were obtained with optical coherence tomography (OCT). Data were acquired in both patients with Fourier-domain (FD) OCT imaging (RTVue-100; Optovue Inc., Fremont, CA). The principles of the method and our recording and analysis techniques have been published [6,10,14,29,37]. For this work, the line protocol of the FD-OCT system, with greater speed of acquisition, was used to obtain 4.5-mm-long scans composed of 1,019 longitudinal reflectivity profiles (LRPs) acquired in approximately 4 ms. Overlapping, nonaveraged, OCT scans were used to produce a digital montage covering up to 9 mm eccentricity from the fovea along the horizontal meridian. A video fundus image was acquired with each OCT scan. Regions of interest in extramacular retina were examined with single horizontal scans; the location and orientation of the scan relative to retinal features (blood vessels, RPE depigmentation, and optic nerve head) were determined by using the digital images of the fundus.

Postacquisition processing of OCT data was performed with custom programs (MATLAB 6.5, MathWorks, Natick, MA). LRPs that composed the OCT scans were aligned by straightening the major RPE reflection [6,10,14,37]. Two nuclear layers, the outer photoreceptor nuclear layer (ONL) and the inner nuclear layer (INL), were defined in regions of scans showing two parallel stereotypical hyporeflective layers sandwiched between the RPE and vitreoretinal interface [6,14,29]. Inner retinal thickness was defined as the distance between the signal transition at the vitreoretinal interface and the sclerad boundary of the INL or the single hyporeflective layer continuous with the INL [6,29,37]. In normal participants, the signal corresponding to the RPE was assumed to be the most sclerad peak within the multi-peaked scattering signal complex [37], deep in the retina. In abnormal retinas, the presumed RPE peak was sometimes the only signal peak deep in the retina; in other cases, it was apposed by other major peaks. In the latter case, the RPE peak was specified manually by considering the properties of the backscattering signal originating from layers vitread and sclerad to it [38]. Also assessed were the presence of photoreceptor inner segment (IS) and outer segment (OS) signal and outer limiting membrane (OLM) signals vitread to the RPE peak [38].

### Pupillometry

The direct transient pupillary light reflex (TPLR) was elicited and recorded as previously published [10,26,39]. In brief, TPLR luminance-response functions were derived from responses to increasing intensities (from −6.6 to 2.3 log scot-cd.s.m^−2^) of green stimuli with short duration (0.1 s) presented monocularly in the dark-adapted state. The light-stimulated pupil was imaged with an infrared-sensitive video camera (LCL-903HS; Watec America Corp., Las Vegas, NV). Images were digitized by two instruments simultaneously: a digital image processor (RK-706PCI ver. 114 3.55; Iscan, Inc., Burlington, MA) sampled the horizontal pupil diameter at 60 Hz; and a video digitizer (PIXCI SV4 board, ver. 2.1; Epix, Inc., Buffalo Grove, IL) produced a computer file of the video sequence. Records were 5.7 s long, with a 1 s prestimulus baseline. Artifacts resulting from blinks were excluded from the analysis. Manual measurement of digitized video images postacquisition complemented automatic measurements performed during the acquisition. TPLR response amplitude was defined as the difference between the pupil diameter at a fixed time (0.9 s) after the onset of the stimulus and the prestimulus baseline. TPLR response threshold was defined as the stimulus luminance that evoked a 0.3 mm criterion (limit of spontaneous oscillations in pupil diameter), [26] amplitude at 0.9 s.

## Results

Two patients were diagnosed clinically with LCA in the first few months of life. Both patients had nystagmus, limited visual responding from about one month of age, and nondetectable electroretinograms. There was no family history of similar visual disorders for either patient and there was no known parental consanguinity. Both patients were of Ashkenazi Jewish ancestry. Molecular screening was performed in the patients for variations in the *AIPL1, CEP290, CRB1, CRX, GUCY2D, RDH12, RPE65* and *RPGRIP1* genes [2]. There were no plausible disease-causing variations in any of these genes. Further studies revealed a homozygous CAG>TAG nucleotide substitution in the coding sequence of the *LCA5* gene, resulting in an amino acid change of Gln279Stop. This *LCA5* mutation has previously been reported in one other patient of Ashkenazi Jewish ancestry [22].

### Clinical features and en face infrared fundus images

**Figure 1:**
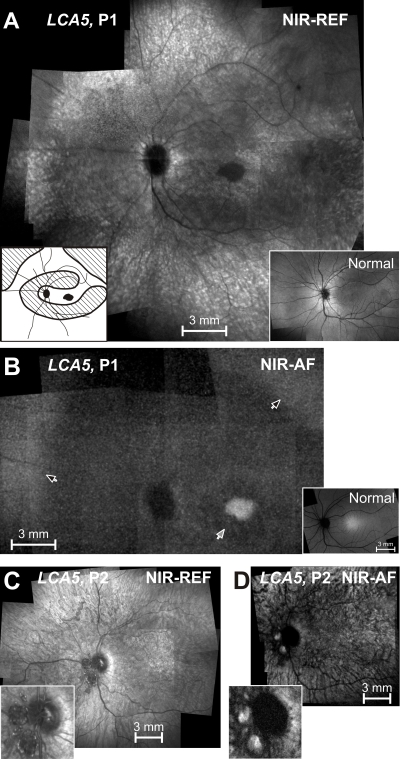
En face near-infrared reflectance and autofluorescence images of the *LCA5* patients. **A:** Near-infrared (NIR) reflectance (REF) image of the left fundus of P1 is shown. Inset to the left is a schematic drawing of the retinal regions corresponding to low reflection (black), intermediate reflection (hatched), and high reflection and choroidal visibility (white). Inset to the right is a NIR reflectance view of the fundus of a 6 year-old child with normal vision. **B:** Near-infrared-autofluorescence (NIR-AF) image of the left fundus of P1 is shown. Black arrows indicate the boundaries of the midperipheral transitions to healthier retinal pigment epithelium; and gray arrow points to the parafoveal annular region of low intensity. Inset is a normal image. **C:** NIR reflectance image of the left fundus of P2 is shown. Inset is an enlarged view of the optic nerve head (ONH) region with ONH drusen. **D:** NIR-AF image of the left fundus of P2 is shown. Inset is an enlarged view of the ONH region. All images are shown contrast stretched for visibility of features.

P1, at age 6 years, had light perception vision, hyperopia (+6.50 sphere), nystagmus, and no corneal or lenticular opacities. An en face montage of the ocular fundus of P1 using NIR reflectance imaging showed a distinctly demarcated dark central island surrounded by alternating elliptical regions of lighter and darker appearance (Figure 1A). A schematic of the darker-appearing regions is shown (Figure 1A, inset left). The reflectance pattern in *LCA5* P1 was in contrast to the more homogeneous NIR reflectance view from an age-matched normal subject (Figure 1A, inset right). The lighter regions in *LCA5* P1 showed greater visibility of the choroid, suggesting depigmentation of RPE. Darker regions likely correspond to more preserved RPE. NIR-AF provided further information on the RPE with the use of melanosome-specific signals from the fundus [31]. High intensity NIR-AF signal originates from the irregular-shaped central island, suggesting a preserved or hyperpigmented RPE at this location (Figure 1B). There was a region of lower intensity NIR-AF in the parafovea, and this likely corresponds to chorioretinal atrophic change. At greater eccentricities, there was an incremental increase in NIR-AF. In the superotemporal and superonasal near midperiphery, there was a distinct boundary of a further increase in NIR-AF signal with a spatially homogeneous appearance, representing more retained and pigmented RPE.

*LCA5* patient 2 (P2) was followed from age 6 months through age 25 years. Visual acuity loss increased from 7/200 (right eye) and 20/400 (left eye) at age 6 years to light perception in both eyes at age 25. A high hyperopic refractive error was present at all visits (+10.00 sphere); there was nystagmus but no corneal or lenticular opacities. Funduscopic examinations throughout the years noted retina-wide granular-appearing pigmentary disturbances and optic disc drusen in both eyes, a finding previously noted in *LCA5* [1]. An en face montage of the ocular fundus of P2 at age 25, using NIR reflectance imaging, showed a light appearance with visibility of the choroid, suggesting depigmentation of RPE (Figure 1C). NIR-AF displayed a choroidal-appearing pattern; specifically there was no evidence of a central region of hyperautofluorescence or peripheral boundary to a relatively increased signal (Figure 1D). The optic disc drusen were evident in the NIR reflectance image (Figure 1C, inset) and they revealed hyperautofluorescence under NIR excitation (Figure 1D, inset). Optic disc drusen are known to show AF under short-wavelength excitation [40], but their NIR-AF signals have not been described.

Between ages 8 and 25 years, P2 was able to perform kinetic perimetry and showed only a central island of perception in each eye of roughly 2–3 degrees in diameter (with a large bright target, V-4e). Static threshold perimetry, using an achromatic target (size V, but 1 log unit brighter than the kinetic perimeter 4e) in the dark-adapted state, was performed in the left eye of P2. This was initially done when the patient was 17 years old and then repeated at 25 years of age. The target was only detected in the central field, and thresholds were elevated by >5.5 log units at both visits [27].

### Cross-sectional retinal imaging: preserved photoreceptors adjacent to retinal disorganization

**Figure 2:**
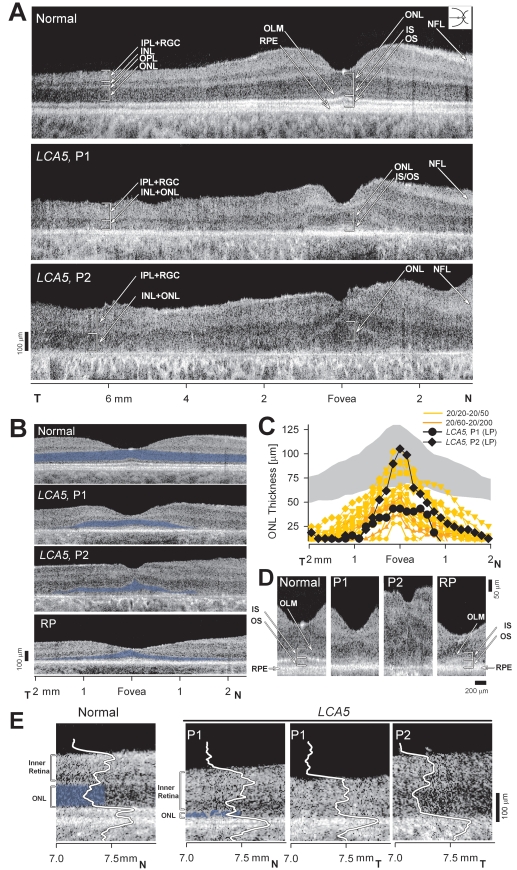
Dysmorphology in the retina of *LCA5*. **A:** Cross-sectional OCT images across the horizontal meridian are shown for a normal 6-year-old subject (upper) and *LCA5*, P1 (middle) and P2 (lower). Layers or structures are labeled as individual or combined laminae. IPL+RGC, inner plexiform and retinal ganglion cell layers; INL, inner nuclear layer; ONL, outer nuclear layer; OLM, outer limiting membrane; IS, inner segments; OS, outer segments; RPE, retinal pigment epithelium. N: Nasal, T: Temporal retina. **B:** Central scans are shown for a normal subject, P1, P2, and an RP patient with a residual and abnormally reduced central island of retinal structure. ONL layer is highlighted in blue. **C:** Photoreceptor nuclear layer thickness horizontally across the central 4 mm of retina is graphically displayed for a group of normal subjects (gray represents mean±2SD; n=26; ages 5–58 years), 19 patients with retinal degeneration but not *LCA5*, and the *LCA5* data. For comparison with *LCA5* P1 and P2 with light perception (LP) vision, the ONL data from the patients with retinal degeneration are color-coded by their visual acuity levels. **D:** Magnified (1.2 mm across) horizontal cross-sections through the fovea of *LCA5* P1 and P2 are compared to those of the 6-year-old normal subject (left panel) and an RP patient (right panel) with similarly reduced foveal ONL. **E:** Cross-sectional, 0.9 mm-long, extramacular images from *LCA5* P1 and P2 are compared to a normal subject. Longitudinal reflectivity profiles (LRP, white traces) overlaid on the scans show signal features corresponding to the different retinal laminae. The ONL is highlighted (blue) next to the corresponding LRP signal feature. *LCA5* P1 (left) at 7 to 7.8 mm in nasal retina shows remnants of ONL, retained retinal lamination and a thickened inner retina (bracketed to the left of the scans) compared to the normal subject at the same eccentricity. Scans from 7 mm in temporal retina from both *LCA5* patients show complete loss of ONL signal and retinal disorganization with a bilaminar appearance of the LRPs.

Retinal laminar architecture of *LCA5* P1 at age 6 years and P2 at 25 years was examined by high resolution cross-sectional OCT imaging (Figure 2). The central 14 mm scan of retina along the horizontal meridian in a normal subject (Figure 2A, upper) illustrates the foveal depression and the hyporeflective and hyperreflective layers that have been shown to have a predictable relationship to histologically defined layers [14,29,37]. Lamination was also present in the patient scans, but there were abnormalities (Figure 2A, middle and lower). Both patients had a foveal depression; this was identified as the deepest pit on raster scanning. P2 had far less depth to the foveal pit than P1; there was notable epiretinal membrane in this scan and in others (data not shown). Unlike the representative normal scan, both patients also showed some tissue vitread to the foveal ONL. The epifoveal tissue in P1 appeared continuous with parafoveal INL and inner plexiform and retinal ganglion cell layers. Foveal ONL thickness in P1 measured 43 µm, which is reduced to about 48% of normal (normal mean±SD=90±8.6 µm; n=5; ages 5–15 years), suggesting loss of foveal cone cells. Foveal ONL thickness in P2 measured 101 µm, which is within normal limits. Whether this hyporeflectivity measured at the fovea in P2 represents only cone nuclei or is a more complex structure with, for example, Müller cell hypertrophy is uncertain [38]. Eccentric to roughly 1.5 mm nasal and temporal to the fovea, the ONL in both patients is barely discernible. At approximately 7 mm eccentricity into the temporal retina, normal lamination is no longer present, and the patient scans have almost a “bilaminar appearance” with a thick vitread hyperreflective layer and a deep thickened hyporeflective layer. The thick superficial layer likely includes inner plexiform layer and retinal ganglion cell layer, and the deeper layer may be an amalgam of thickened INL with remnant photoreceptor nuclei [16,29]. Retinal thickness and especially inner retinal thickness is remarkably greater in P2 than in P1.

We determined whether the residual foveal ONL in the *LCA5* patients was expected to be associated with light perception vision. In vivo histopathology of the *LCA5* patients was compared to a group of patients with retinal degeneration and similar foveal ONL thickness, and who had been found, by dark-adapted chromatic perimetry, to be at a stage of disease that had reduced vision to a central island with only abnormal cone function [41] (Figure 2B-D).

The 19 patients with retinal degeneration compared with *LCA5* patients were in the general clinical category of retinitis pigmentosa (RP) and showed a range of visual acuities from 20/20 to 20/200. These patients were 21 to 59 years of age with diagnoses that included X-linked RP due to *RPGR* mutations, Usher syndrome 1B and 2A, and ungenotyped RP. Their ONL profiles were similar to those of the *LCA5* patients (Figure 2C). This suggested that the foveal cone cells in the *LCA5* patients were not functioning optimally.

Magnified views of the foveal center of P1 and P2 (Figure 2D) demonstrate that the IS/OS signal appears ill-defined in P1 or nearly not discernible in P2. For comparison are similar views of the foveal center for a normal subject and for an RP patient. The RP patient has thinned ONL but more definable IS/OS signal and better foveal function (visual acuity, 20/40). This abnormal signal deep to the foveal cone ONL may indicate structural abnormalities in the cone IS/OS, possibly a consequence of a ciliopathy, with resultant visual dysfunction. At greater eccentricities there was severe ONL reduction. Remnants of ONL observed in the nasal retina of P1 were accompanied by a thickened inner retina (Figure 2E, left P1 panel) suggestive of retinal remodeling. Total photoreceptor layer loss in both patients resulted in a disorganized retinal structure with a bilaminar appearance (Figure 2E, right two panels) with a hyper-thick (P1=148 µm; P2=212 µm) inner retina (normal mean±SD=108±20 µm).

### Pupillometric abnormalities in *LCA5*

**Figure 3:**
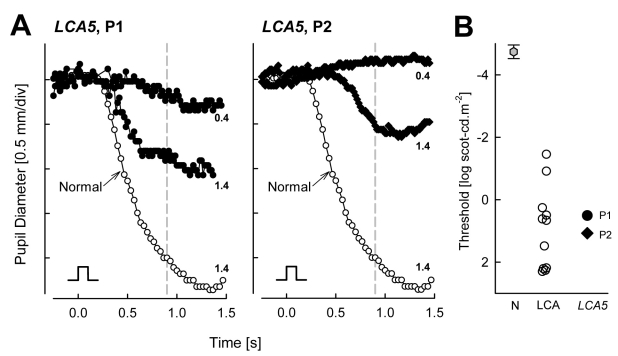
Transient pupillary light reflex (TPLR) abnormalities in *LCA5* patients. **A:** Change in pupil diameter is plotted as a function of time in response to short duration (0.1 s) light stimuli of two different intensities (0.4 and 1.4 log scot-cd.s.m^−2^; denoted at right end of traces) in the *LCA5* patients (filled symbols). A response elicited with the brighter stimulus in a 9-year-old normal subject is shown for comparison (unfilled symbols). Stimulus monitor is shown at lower left of each of the two panels. **B:** TPLR response thresholds to a 0.3 mm criterion response in the *LCA5* patients (filled symbols) are compared to a group of 11 LCA patients, age 1–58 years, with severely impaired vision (unfilled symbols). TPLR response amplitudes are measured at a fixed time of 0.9s (vertical dashed line in **A**). Thresholds in the *LCA5* patients and other LCA patients show elevations in excess of 5 log units from normal. Gray hexagon denotes normal mean±2SD.

TPLR was used to quantify objectively the pupillometric sensitivity to light in these two *LCA5* patients with limited vision and nondetectable electroretinograms. For dark-adapted normal eyes, a criterion response (0.3 mm contraction at 0.9 s) of the TPLR is near −5.0 log scot-cd.s.m^−2^ [10,26]. The amplitude of the normal TPLR response grows as a function of stimulus intensity reaching a saturation value of approximately 2 mm by 2 log scot-cd.s.m^−2^. Both *LCA5* patients showed marked abnormalities in TPLR. Responses for the patients were first detectable with higher intensity stimuli. P1’s response was 0.22 mm at 0.4 log scot-cd.s.m^−2^; and P2’s response was 0.55 mm at 1.4 log scot-cd.s.m^−2^. Contraction amplitude to the brighter flash was reduced to 51% of normal mean (1.91±0.40 mm, n=12) in P1 and to 29% of normal mean in P2 (Figure 3A). TPLR thresholds derived from intensity response functions were elevated by 5–6 log units in the *LCA5* patients (P1=0.51, and P2=1.08 log scot-cd.s.m^−2^) compared to normal subjects (mean±2SD=-4.74±0.22 log scot-cd.s.m^−2^, n=12).

The TPLR threshold abnormalities in *LCA5* were similar in order of magnitude to those observed in a group of eleven patients, ages 1–58 years, with different molecular causes of LCA and comparable levels of visual dysfunction (visual acuities of 20/800 or worse). The latter group of LCA patients had TPLR thresholds with mean±2SD of 0.78±2.40 log scot-cd.s.m^−2^ (Figure 3B).

## Discussion

Clinical features shared by most of the *LCA5* patients reported to date include the following: severe visual disturbances and nystagmus from birth, hyperopia, nondetectable electroretinograms, and a spectrum of ophthalmoscopic findings from near normal appearance in infancy to pigmentary retinopathy [20-22,24,25]. Macular atrophy was noted in older members of an *LCA5* family in which younger members showed minimal change [21]. Maculopathy has also been observed in early stages of the disease in other *LCA5* patients [24]. The studies of photoreceptor and RPE integrity in the *LCA5* patients of this study extend the previous reports.

The presence of a foveal depression with apparently retained foveal ONL suggests that central retinal development occurred to some degree in the *LCA5* patients [42,43]. However, the foveal architecture was not entirely normal: there were visible laminae vitread to the foveal ONL in P1 and thickening of the fovea in P2, the latter being previously noted in choroideremia [38]. The exact basis of these observations is uncertain but could be due, for example, to incomplete migration of the inner retina toward the periphery during foveal development [42,43], epiretinal membrane distortion of foveal structure, or Müller cell activation, hypertrophy or proliferation in response to photoreceptor cell death [38]. Assuming there are retained foveal cone photoreceptors in *LCA5*, then the comparison of foveal ONL thickness with other retinal degenerations at similar severity levels by OCT structure suggests that *LCA5* visual acuity was far worse than that in the others. This may in part be due to dysfunction from a ciliopathy with disrupted protein transport between inner and outer segments [44]. Deep subfoveal retinal structure in the *LCA5* patients, however, was definitely abnormal in the region conventionally attributed to photoreceptor IS, cilia, and OS, suggesting a pathological component. Additional study of the outer retina of *LCA5* with ultrahigh resolution OCT would seem valuable [45,46]. The low visual acuity at a young age may result from cone photoreceptor ciliopathy but a component of refractive amblyopia also cannot be ruled out.

A diminished photoreceptor layer was detected at extra-central locations, but regions of no detectable ONL were also present. This indicates that even as early as age 6 years, there is considerable photoreceptor loss. Between loci with detectable ONL were regions that had disorganized lamination. The hallmarks of retinal remodeling were present in these regions: thickened inner retinal layers including inner plexiform and nuclear layers, and reduced to imperceptible outer plexiform and outer nuclear layers [16,29,38,47]. We previously compared such OCT findings in human *CEP290*-LCA and adRP due to *rhodopsin* mutations with histopathology of relevant murine models (*rd16* mouse and T17M *rhodopsin* mutant mouse) and the results of these studies support the notion of delaminated remodeled retina in the *LCA5* patients [16,29].

The pattern of pigment preservation and loss, as revealed by NIR reflectance and AF in P1, is worthy of comment. If pigmentary losses are assumed to be a sequela of photoreceptor losses, as in many other retinal degenerations [29,48], we speculate that the preserved pigment could be a useful surrogate for photoreceptor preservation. The patterns of alternating elliptical-appearing areas of decreased and increased pigmentation are reminiscent of topographical maps of human photoreceptor density [49]. The preserved central photoreceptors and pigment may be simply due to the high density of cones in this region; the ellipse of increased pigmentation near the vessel arcades may represent relative preservation of pigment and photoreceptors due to the higher density of rod photoreceptors in that region. The decreased pigment between these two regions may be due to the drop of receptor density (combined rod and cone) between foveal cone density peak and rod density peak. A more peripheral ring of increased cone density has been documented and may relate to the increased pigment noted in the *LCA5* patient [49].

In the current work, the TPLR proved to be a helpful adjunct measure of vision in these *LCA5* patients with severe visual loss. Pupils responded to short-duration, bright light stimuli, optimized to elicit a pupillary reflex mediated by conventional photoreceptors [26] although a contribution from intrinsically light-sensitive ganglion cells cannot be ruled out [50]. The TPLR was abnormal in the patients, as has been described in other groups of LCA patients [10,26,39]. Thresholds were extremely elevated as in other LCA patients with comparable degrees of visual loss.

Once animal models of *LCA5* are established and investigated, it will be of strong interest to decide how faithfully they will relate to the noninvasive retinal structural and functional findings in *LCA5* patients. The major rod loss at an early age, evident already in the 6-year-old *LCA5* patient of this study, should be considered even at the proof-of-concept stage of study in models of *LCA5*. It may be of value eventually to direct therapy at cones, whether by gene replacement or other methods. Our studies of the *CEP290* form of LCA, another ciliopathy with mainly a residual central island of cones and RPE, led to a similar recommendation [16].
